# Complex germline and somatic mutation processes at a haploid human minisatellite shown by single-molecule analysis

**DOI:** 10.1016/j.mrfmmm.2008.09.008

**Published:** 2008-12-15

**Authors:** Morag E. Shanks, Celia A. May, Yuri E. Dubrova, Patricia Balaresque, Zoë H. Rosser, Susan M. Adams, Mark A. Jobling

**Affiliations:** Department of Genetics, University of Leicester, University Road, Leicester LE1 7RH, UK

**Keywords:** Y chromosome, Minisatellite, MSY1, Germline mutation, Somatic mutation, Gene conversion

## Abstract

Mutation at most human minisatellites is driven by complex interallelic processes that give rise to a high degree of length polymorphism and internal structural variation. MSY1, the only highly variable minisatellite on the non-recombining region of the Y chromosome, is constitutively haploid and therefore precluded from interallelic interactions, yet maintains high diversity in both length and structure. To investigate the basis of its mutation processes, an unbiased structural analysis of >500 single-molecule MSY1 PCR products from matched sperm and blood samples from a single donor was undertaken. The overall mutation frequencies in sperm and blood DNAs were not significantly different, at 2.68% and 1.88%, respectively. Sperm DNA showed significantly more length mutants than blood DNA, with mutants in both tissues involving small-scale (1–3 repeat units in a 77 repeat progenitor allele) increases or decreases in repeat block lengths, with no gain or loss bias. Isometric mutations altering structure but not length were found in both tissues, and involved either the apparent shift of a boundary between repeat unit blocks (a ‘boundary switch’) or the conversion of a repeat within a block to a different repeat type (‘modular structure’ mutant). There was a significant excess of boundary switch mutants and deficit of modular structure mutants in sperm. A comparison of mutant structures with phylogenetically matched alleles in population samples showed that alleles with structures resembling the blood mutants were unlikely to arise in populations. Mutation seems likely to involve gene conversion via synthesis-dependent strand annealing, and the blood-sperm differences may reflect more relaxed constraint on sister chromatid alignment in blood.

## 1 Introduction

Human minisatellites, tandem arrays of repeat units between 9 bp and 100 bp in length, owe their spectacular degrees of allele length polymorphism to largely interallelic processes in the germline that generate novel alleles following non-reciprocal exchange processes [1]. Internal allele structures, defined by the patterns of variant repeat units assessed via minisatellite variant repeat PCR [2] (MVR-PCR), are also highly diverse, and determining such structures allows a fine-scale picture of mutation processes to be obtained. Studies of sperm DNA have provided detailed information about male germline mutation, and comparative studies in blood DNA have shown that the pathways of mutation in the germline and soma are distinct [3–6]. Somatic processes are slower and simpler than those in the germline, with a predominance of intra-allelic mechanisms.

The mapping of most minisatellites to the recombinationally active termini of human chromosomes [7], and the coincidence of the mutationally active ends of some minisatellites with known recombination hotspots [8] and with motifs associated with hotspot activity [9], suggests that the majority of these loci arise as by-products of localised meiotic recombination. An observation consistent with this idea is the paucity of polymorphic minisatellites on the constitutionally haploid non-recombining region of the Y chromosome [10]. There are only two known examples: one, MSY2 [11], barely qualifies as a minisatellite, with a mere two distinct alleles (of 3 and 4 repeat units) described; in contrast, the other, MSY1 (*DYF155S1*) [12,13], displays length polymorphism of 48–118 repeat units and considerable internal structural diversity, with a virtual heterozygosity of 99.9%.

Despite its high degree of polymorphism, MSY1 is very different from the ‘classical’ minisatellites that are detected in traditional DNA fingerprinting experiments and linked to meiotic recombination processes. While the latter are GC-rich loci, MSY1 is 75% A + T [12]. Its internal allele structures, defined by the distribution of several base-substitutional variants of a basic 25 bp repeat unit, are simple: unlike the highly interspersed structures of many autosomal loci, variant repeats in MSY1 alleles are organised in blocks. The repeat unit is predicted to form a hairpin, and the likely involvement of this putative secondary structure in mutation is supported by the fact that repeat units of variant (non-25 bp) lengths are never observed.

The mutation mechanisms that maintain such high variability despite the straitjacket of constitutive haploidy are of considerable interest: although diploid minisatellites may be largely driven by interactions between alleles, intra-allelic processes are also active, and studying events on the Y chromosome allows exclusive access to these.

There have been a number of previous studies providing information about MSY1 mutation: inferences from diversity suggested a mutation rate to alleles of different structure of 2–11% per generation [12]. A study of alleles in deep-rooting pedigrees [14] yielded a mutation rate of ∼3% [15], and suggested that changes in the structure of an allele without changes in its length (‘isometric’ mutation) could occur. This was supported in a study of MSY1 allele transmissions in 1071 father–son pairs [16], which gave an overall mutation rate of 3.8%.

No study, however, has been able to observe the spectrum of mutation events arising from a single progenitor allele structure, or to compare the processes at work in the germline and soma. Here, we describe an unbiased study of mutants arising in sperm and blood DNA from a simple progenitor allele structure in a single donor. Overall mutation frequencies are 2.68% and 1.88%, respectively. Structures of mutant alleles in blood DNA are markedly different from those in sperm, and phylogenetic analysis of allele diversity suggests that they are unlikely to arise in populations, pointing to distinct germline and somatic pathways of mutation.

## 2 Materials and methods

### 2.1 Preparation of DNA

Red blood cells in a 200 μl sample from an anonymous donor were lysed using 1× SSC, and the white cell pellet digested using 2 μg/ml proteinase K in 1% SDS. Following phenol/chloroform extraction, DNA was recovered by ethanol precipitation. Sperm DNA from the same donor was extracted as described [17]. DNA concentrations were estimated by comparison with standards after gel electrophoresis, and diluted to ∼5 ng/μl.

### 2.2 Single-molecule PCR amplification of MSY1

Eight 10 μl PCR reactions were set up for each of six notional inputs (100 pg, 50 pg, 20 pg, 10 pg, 5 pg and 2pg) using the external flanking primers SM1 (5′-CTA CAA CAT TAG CAG GAT ATG C-3′) and SM2 (5′-GAG GTT GTT GTG ACT ACA GAT-3′) at 0.3 μM, with PCR buffer [18], 0.5U *Taq* polymerase and 0.025U *Pfu* polymerase. Amplification was to sub-visible level in order to avoid contamination problems, under the following conditions: 95 °C for 1 minute, 62 °C for 3 min and 68 °C for 3 min for 12 cycles. To detect positive reactions, a secondary PCR reaction was carried out using nested flanking primers. A 1 μl aliquot of the primary PCR product was amplified with standard MSY1 flanking primers [12], Y1A^+^ (5′-ACA GAG GTA GAT GCT GAA GCG GTA TAG C-3′) and Y1B^+^ (5′-GCA ACT CAA GCT AGG ACA AAG GGA AAG G-3′) each at 0.3 μM under the above conditions for 16 cycles, prior to gel electrophoresis and detection of DNA by ethidium bromide staining.

Single-molecule amplification is considered to be achieved when approximately 50% of the reactions are negative. The input volume of the set of eight reactions fulfilling this condition provided the required input volume for the subsequent single-molecule experiments. For each experiment 40 matched sperm and blood PCR reactions, for both primary and secondary amplifications, were carried out.

### 2.3 Structural analysis of single-molecule products

To identify positive reactions, secondary PCR products were resolved on a 20 cm 1% (w/v) agarose gel in 1× TBE. Secondary PCR was repeated on all positive reactions, using the primary PCR product as template, followed by resolution on a 40 cm 1% (w/v) agarose gel at 120 V for ∼48 h to allow detection of length variants to single-repeat-unit resolution.

**Fig. 1:**
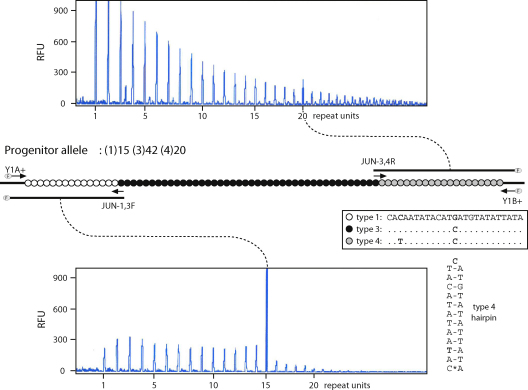
Repeat type, structure of progenitor MSY1 array, and junction primer strategy for mapping mutants. In the middle is shown a schematic structure of the donor allele, with repeat units indicated by circles and sequences given in the key. Arrows indicate primers. Below and above are shown electropherograms showing, respectively the results of typing the 1, 3 and 3, 4 repeat unit boundaries, using primer combinations Y1A^+^/JUN-1, 3 and Y1B^+^/JUN-3, 4. Junction primers are fluorescently labelled (‘F’). RFU: relative fluorescent units. The junction primers are directed at the boundaries, but also yield PCR products corresponding to other local repeats through mispriming. The putative hairpin adopted by a type 4 repeat is also shown.

The progenitor array structure was determined using a radioactive MVR-PCR technique [12]. Internal structures of all single-molecule products were defined using primers targeted at the junctions between blocks of repeat types [19] (Fig. 1), paired with flanking primers 5′-labelled with 6-FAM. Primer JUN-1,3F (5′-CGC TGC CAA CTA CCG CAC ATG TAT ACA TGA TGT ATA TTG TGT ATA ATA TAC ATC ATG TAT ATT G-3′) was specific to the type 1/type 3 junction, and paired with Y1A^+^; and primer JUN-3,4R (5′-CGC TGC CAA CTA CCG CAC ATG CAC AAT ATA CAT CAT GTA TAT TAT ACA TAA TAT ACA TC-3′) was specific to the type 3/type 4 junction, and paired with Y1B^+^. Reactions contained Amplitaq Gold buffer (Applied Biosystems), 1.5 mM MgCl_2_, 1 μg/ml BSA (NEBL), 0.2 mM dNTPs, 0.04U Amplitaq Gold (Applied Biosystems), and 1 μM each primer, together with 1 μl primary PCR product. General PCR conditions were: 95 °C for 11 min, followed by 95 °C for 1 min, 65 °C for 3.5 min and 72 °C for 5 min for 35 cycles. Products were resolved on an ABI3100 Genetic Analyzer and sizes determined with reference to a ROX-400 standard (Applied Biosystems).

Structures of putative mutants involving alteration to the blocks of type 1 or type 4 repeats were confirmed by conventional MVR-PCR.

### 2.4 Determination of MSY1 allele diversity within haplogroup R1b3

The donor’s Y chromosome was classified into haplogroup R1b3 [20] by binary marker typing of the marker M269 [21] as described [22]. A collection of MSY1 codes from a set of 159 hgR1b3 chromosomes was compiled using standard MVR-PCR [12].

### 2.5 Estimation of mutation frequencies

The mean number of amplifiable molecules in each initial input was estimated from the Poisson distribution [23] using the equation *z* = e^−m^, where *z* is the frequency of negative PCR reactions, implemented in a program that allows for the variance that exists between different experimental replicates, resulting from uncertainty in the number of amplifiable molecules.

The frequencies of minisatellite mutation, 95% confidence intervals and standard errors were estimated using a modified approach proposed by Chakraborty and co-workers [23]. A *t*-test was used to compare blood and sperm mutation frequencies after Poisson analysis.

## 3 Results

To investigate mutation at MSY1 we recruited a donor to provide matched sperm and blood samples who carried an MSY1 array of typical overall length (77 repeats) with the internal structure of (1)15 (3)42 (4)20 (Fig. 1), as determined by traditional MVR-PCR. This array belongs to the simplest modular structural class, denoted as ‘1, 3, 4’ – a block of type 3 repeats, flanked by blocks of type 1 and type 4 repeats. Binary marker typing showed that the donor’s Y chromosome belongs to the prominent western European lineage haplogroup R1b3.

Evidence from previous pedigree studies [15,16] and phylogenetic analysis [12,13,24–26] suggests that mutations that alter the structure of alleles, but not their overall array length (‘isometric’ mutations) may be common at MSY1. A thorough survey of mutation at this locus therefore requires structural analysis of a sizeable population of single-molecule-derived PCR products, including those showing no length alteration.

Sperm and blood DNA were extracted and diluted to single-molecule level, and then underwent PCR as described in Section 2. A series of 24 experiments, each containing 40 sperm DNA and 40 blood DNA reactions, were carried out.

### 3.1 Sperm mutants

**Fig. 2:**
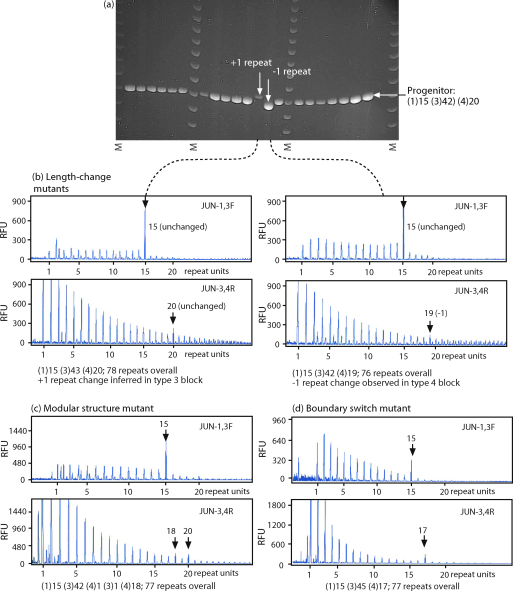
Detection of mutants by flanking and junction PCR. (a) Example of an agarose gel, showing +1 and −1 repeat length mutants in sperm DNA. The size marker (‘M’) is 100 bp ladder (Promega). (b) Electropherograms showing the structures of length mutants. RFU: relative fluorescent units. Junction products are shown by short vertical arrows, with the number of repeat units indicated. (c) Electropherograms showing an example of a modular structural mutant. Note that this blood mutant is isometric, retaining a length of 77 repeat units. (d) Electropherograms showing an example of a boundary switch mutant. Note that this sperm mutant is isometric, retaining a length of 77 repeat units.

**Fig. 3:**
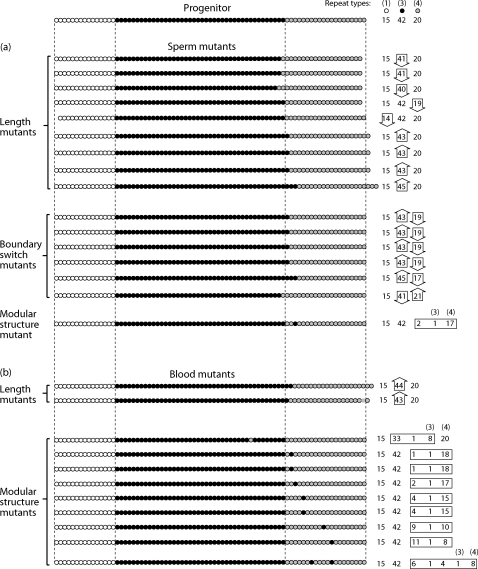
Structures of mutant alleles. At the top is shown the progenitor structure, with circles corresponding to repeat units (see Fig. 1), and a simplified structure to the right. (a) Mutants identified in sperm DNA. Showing length mutants, boundary switch mutants, and the single example of a boundary switch mutant. Large open arrows to the right indicate gains or losses of repeats with respect to the progenitor. (b) Mutants identified in blood DNA. Showing length mutants, and multiple modular structural mutants; note the absence of boundary switch mutants.

Initial experiments sought to identify length mutants. From the 24 sperm DNA experiments 597 molecules were amplified using nested PCR and a total of 9 mutants observed as PCR products larger or smaller than the progenitor allele (Fig. 2(a)). This corresponds to a mutation frequency to new-length alleles of 1.51% (9/597 amplifiable molecules). There was no preference for gain or loss of repeats, with four mutants representing gains, and five losses (Fig. 3(a)). All mutants were within three repeats of the original progenitor size.

Using a fluorescent typing system the positions of repeat block junctions were mapped within each array, thus counting the numbers of repeats within the blocks of type 1 and type 4 repeats (Fig. 2(b)). This allowed the structure of mutants to be determined: if no change was evident in the type 1 or type 4 blocks of repeats, but an overall size alteration had occurred, the mutation must by elimination lie within the central block of type 3 repeats. Seven of the nine length mutations were in the latter category, and one lay in each of the flanking blocks of type 1 and type 4 repeats (Fig. 3(a)). These proportions do not depart significantly from expectation (*p* > 0.05; chi-square test), given the proportion of the array occupied by each block.

To identify isometric mutants in sperm DNA, junction-mapping PCR was carried out on all 588 single-molecule products showing no overall allele length alteration (Fig. 2(c) and (d)). If a change was observed in the length of the type 1 or type 4 repeat block, taken with the overall conservation of array length this would imply that a simple compensatory change had occurred within the central block of type 3 repeats. Using this approach two different types of isometric mutations were observed – simple mutations involving no modular structural change (Fig. 2(d)), and complex mutations in which length was conserved, but the modular structure was altered (Fig. 2(c)).

Six simple isometric mutants (Fig. 3(a)) had the same overall number of repeats in total but the numbers of repeats in the three individual blocks varied, *e.g.* from the progenitor structure of (1)15 (3)42 (4)20 to (1)15 (3)43 (4)19. This mutation type, in which the gain of one or more repeats in one block is accompanied by the loss of the same number of repeats from an adjacent block has been termed a ‘boundary switch’ [15], since it appears as if the boundary between repeat blocks shifts along the array. All boundary switch events observed involved the adjacent blocks of repeat types 3 and type 4, and five out of six involved the loss of type 4 repeats coupled with the gain of type 3 repeats. The largest scale boundary switch events involved three repeats.

One complex isometric sperm mutation involves an alteration in the modular structure of the array (Fig. 3(a)) – the structure changes from 1, 3, 4 to 1, 3, 4, 3, 4. The blocks of type 1 and central type 3 repeats are unaltered, but one repeat within the block of type 4 repeats has apparently changed into a type 3 repeat. This event, like the simple boundary switch, involved an alteration at the boundary of the type 3 and type 4 repeats.

The observed isometric sperm mutations are thus non-uniformly distributed along the MSY1 array, with all seven involving changes within the type 4 block, and none involving the type 1 block. While this suggests a polarity towards the type 4 end of the array, the differences between the two ends of the array are not statistically significant (*p* > 0.05; chi-square test).

### 3.2 Blood mutants

Corresponding mutation analyses were then undertaken in blood DNA. Here, only two length mutants were observed in 531 amplifiable molecules (0.38%), involving gains of either one or two type 3 repeats (Fig. 3(b)).

Determination of the structures of the remaining 529 single-molecule PCR products yielded nine isometric mutants, representing a similar frequency to that found in sperm DNA (7/597). However, the underlying structures of these mutants differed markedly from the sperm DNA mutants: there were no instances of simple boundary switches, and all involved a change in modular structure (Fig. 3(b)).

As in sperm DNA, none of the isometric mutants involve alterations to the block of type 1 repeats. Seven of the nine mutants, like the one complex example seen in sperm DNA, involve the apparent change of a single type 4 repeat into a type 3 repeat. In one further case there are two such repeat changes, separated by four unchanged type 4 repeats. In the last mutant, a single type 3 repeat is changed into a type 4 repeat.

Table 1 shows the frequencies of different categories of mutation events in sperm and blood, together with 95% confidence intervals. There is no significant difference in the total of number of mutation events between the two tissues (*t*-test: *p* = 0.375). However, if the length change mutations are considered, then the difference is significant (*p* = 0.049), although observation of only one more mutation within blood would alter this. Considering isometric mutations as a combined class, the difference between blood and sperm DNA is non-significant (*p* = 0.472); however, when this class is divided into boundary switches and modular structural changes, the difference between blood and sperm DNA is significant for the modular mutant class (*p* = 0.016). It was not possible to compare the boundary switch class using a *t*-test, as no events in this class were observed in blood; however, using the approximation of the chi-square test, the difference is significant (chi = 5.37; *p* = 0.025).

### 3.3 Mutants in their phylogenetic context

The natural diversity of MSY1 allele structures found in populations should reflect the germline processes at work, allowing us to ask if the somatic processes we observe really are unusual. To provide a context in which to consider the mutants, we compiled a set of alleles from chromosomes belonging to the same haplogroup as the donor, R1b3, which are all derived by mutation from a common ancestor. Of the 159 alleles (Supplementary Table 1), 145 (91%) have the modular structure 1, 3, 4, with mean allele length ∼73 repeats, and standard deviation ∼3 repeats. Corresponding values for the three individual block lengths are: type 1 – mean ∼ 16, S.D. ∼ 1; type 3 – mean ∼ 39, S.D. ∼ 3; type 4 – mean ∼ 18, S.D. ∼ 2. This predominance of a single modular structure and the tight distributions of lengths of the overall array and of individual blocks attest to the rarity of mutations that alter length or structure radically in the germline, which is consistent with our observations of sperm DNA mutants.

**Fig. 4:**
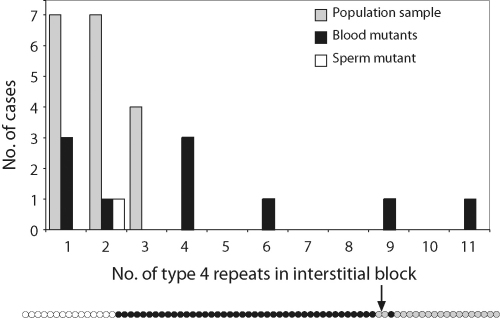
Distribution of the length of the interstitial type 4 repeat block in population samples and mutants.

While each of the mutants represents a unique and independent event, the population samples are the result of successive mutation processes, and subsets of them are likely to be relatively closely related, carrying structural features that are identical by descent. This makes a fair comparison between the alleles in the population and the blood and sperm mutants difficult. However, with this caveat in mind, there are 14 alleles in the population sample that have non-1, 3 ,4 structures (Supplementary Table), and can be compared with the modular structural mutants. In the population sample, and in the one example of a sperm mutant, the interstitial block (or blocks) of type 4 repeats is between one and three repeat units in length. However, among the 10 examples of such blocks in the blood mutants, six are ≥4 repeats in length (Fig. 4), suggesting that the somatic processes giving rise to these mutants are qualitatively different from those underlying germline mutation.

## 4 Discussion

Previous studies of sperm mutation at minisatellites have focused on events that detectably alter allele length [1]. Not only does this ignore isometric events, but it can also exclude gains or losses of small numbers of repeat units, since these are not generally electrophoretically resolved from the progenitor allele. Our study is atypical in providing a complete and unbiased assessment of the mutational spectrum at a minisatellite, regardless of allele length change. Furthermore, because the minisatellite we have studied lies on the non-recombining region of the Y chromosome and is therefore male-specific, an analysis in sperm DNA provides a full picture of mutation, unlike similar analyses at autosomal or X-linked minisatellites, which inevitably neglect events in the female germline.

What evidence is there that the variant alleles we observe are true mutants rather than PCR artefacts? In studies of autosomal minisatellites, the very much lower mutation frequency of somatic compared to germline mutation [4,6] allows blood DNA to act as a natural control for the validation of sperm mutants. In the case of MSY1, however, we observe similar mutation frequencies in both tissues, so this does not apply. Validity of the mutants is suggested by several lines of evidence: (i) while the overall mutation frequency did not differ between the tissues, the structures of the variant alleles are systematically and significantly different in blood and sperm DNA. Such a difference cannot be accounted for by PCR-based processes, and indicates that the mutation analysis is detecting a genuine biological distinction; (ii) structures of variant alleles arising in sperm DNA are consistent with the processes observed in pedigree analysis [15,16], and suggested by phylogenetic analysis of MSY1 diversity [12,13]; (iii) PCR artefacts should have the effect of elevating the apparent mutation frequency observed in sperm DNA, yet (as discussed below) the observed frequency was actually somewhat lower than estimated in independent studies [15,16]; (iv) in each reaction where a variant allele or an isometric mutant was detected, internal structural analysis showed the presence of a single unique amplified molecule, while if artefacts were arising during PCR, we would expect to observe mixed species of molecules; (v) although the suggested hairpin-forming ability of the MSY1 repeat unit might be important in the mutation process under physiological conditions, under PCR conditions where the temperature does not fall below 62 °C it seems unlikely that this AT-rich structure is responsible for slippage-like processes. In any case, such processes would be expected to lead to large deletions within alleles [4], which are not observed.

We can compare our results with previous studies that have given information about MSY1 germline mutation. One inferred mutation rates by analysing MSY1 structures in the descendants of deep-rooting pedigrees [15], assuming that a difference between a pair of descendants was due to a single mutation event, rather than successive events in different generations. The average rate from this study was ∼3%. A second study analysed MSY1 transmission in 1071 father–son pairs [16], thus providing complete ascertainment of mutations, albeit in a diverse collection of chromosomes from different lineages, and with different MSY1 progenitor allele structures; this gave an overall mutation rate of ∼3.8%. Average estimates are therefore similar in all three studies. Rate estimates for the different mutation sub-classes are also similar: isometric mutations are found at 1.3% for the father–son study, 1.7% for the deep-rooting pedigree study, and 1.17% for the current single-molecule study. All three studies are thus consistent in their pictures of MSY1 germline mutation in rates, mutation types, and also a lack of preference for gain or loss events in length mutation.

Our observations of mutants in blood DNA, however, are novel. The similarity of mutation frequencies in blood and sperm DNA contrasts with the situation for many autosomal minisatellites – where they have been accurately measured, somatic processes are generally 100–200-fold slower than those in the germline [4,6]. This may not be surprising, given that rapid autosomal germline mutation is dominated by interallelic events that are precluded for MSY1. There is, however, a possible ascertainment bias in that >80% of the blood mutants observed in our study are isometric, and so would not be observed in studies that focus on length change (usually of ≥ 2 repeats) as a criterion for mutant alleles.

The structures of MSY1 blood mutants are markedly more complex than those found in sperm, with an absence of boundary switch mutants and an excess of modular structural mutants. This, together with the evidence from the population diversity of germline-derived MSY1 alleles, strongly suggests that there are differences in mutation mechanisms at this minisatellite between germline and soma. We can compare these germline/soma differences compare with those seen in specifically intra-allelic processes at autosomal minisatellites (although a fair comparison is difficult because of the ascertainment differences described above). In the case of MS32 [4], all blood mutants are apparently intra-allelic, and 87–97% represent simple deletions or duplications; by contrast, only 54% of intra-allelic sperm mutations are simple, with the remaining examples complex and difficult to interpret. Likewise, for CEB1 [6] blood mutants are again all intra-allelic, with a preponderance of simple deletions and duplications (88%); in sperm, all clearly intra-allelic events involve gains of repeats, and only 15% are simple, with the remainder highly complex. It is therefore possible that the intra-allelic behaviour of MS32 and CEB1, showing much simpler mutation in blood than in sperm, differs fundamentally from that of MSY1.

What molecular mechanisms underlie the mutation events we have observed? The predicted hairpin that can form in one or several adjacent repeats seems likely to play a role. In principle, a cruciform structure could form within a sister chromatid when each strand of a repeat unit (or units) folds into a hairpin; such a cruciform would contain mismatches that could be repaired, leading to repeat type change. However, a consideration of the mismatched base pairs for various combinations of adjacent repeat types suggests that such a mechanism is unlikely, as it would give rise to improbable repeat types. For example, a cruciform structure forming at the junction of blocks of type 1 and type 3 repeats could give rise, following repair, to a type 2 repeat, which has never been observed in that structural context. However, hairpin formation in transiently single-stranded DNA could lead to misalignment of strands and the opportunity for slippage. This is a plausible mechanism for simple changes in allele length, but it cannot easily explain the isometric events we observe.

**Fig. 5:**
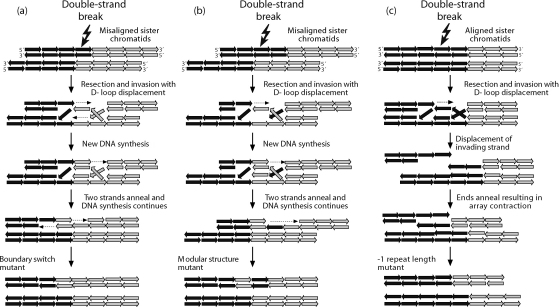
Synthesis-dependent strand annealing as a candidate mechanism for MSY1 mutation. Boundary switch mutant arising from sister chromatids misaligned by a single-repeat-unit. Modular structural mutant arising from sister chromatids misaligned by two repeat units. Example of a length mutant arising from aligned sister chromatids. Open arrows indicate repeat units (black: type 3; grey: type 4); dashed arrows indicate DNA synthesis.

Synthesis-dependent strand annealing (SDSA) [27] is a gene conversion mechanism that has been proposed to explain mutation at GC-rich autosomal minisatellites, including MS32, MS205 and CEB1 [3,6,17]. This mechanism, acting between sister chromatids, could be responsible for the more complex events observed in MSY1 mutation (Fig. 5). The first step is a double-strand break – a lesion that might be promoted by replication fork stalling [28], possibly through the formation of cruciform structures within the array. Following resection, a strand from one chromatid invades the other, thereby creating a D-loop. After DNA synthesis and resolution, the result is the unidirectional transfer of sequence information from one chromatid to another. The outcome, in terms of array change, depends on the initial register of alignment of the sister chromatids. If they are misaligned by one repeat unit (Fig. 5(a)), then a boundary switch mutation can result; if misalignment is by more than one repeat unit, then a modular structural change can occur (Fig. 5(b)). The general observation that modular structural mutants involve repeat type switching of only single-repeat-units suggests that the scale of these conversion events must be restricted (≤25 bp). The position of the converted repeat is dependent on the extent of sister chromatid misalignment; the difference between blood and sperm DNA can then be interpreted as a relaxation of the alignment in the former, allowing conversion events to occur deeper within the blocks of type 3 and type 4 repeats. SDSA can also be invoked to explain length change mutants (Fig. 5(c)). Differences, discussed above, between MSY1 and MS32/CEB1 in germline and somatic intra-allelic mutation behaviour may indicate that the relaxation of sister chromatid exchange, we infer in blood, may not be a general phenomenon, but region- or locus-specific.

The Y chromosome’s non-recombining nature means that all sequences on any Y chromosome share an identical evolutionary trajectory, so a phylogenetic approach to mutation processes is useful [29–31]. Here, we have used the natural diversity of MSY1 alleles within the haplogroup to which our donor’s chromosome belongs, to interpret the diversity of mutants in germline and soma. In a broader context, a detailed study of MSY1 allele diversity within the phylogenetic framework promises to offer insights into rare events and slower processes of mutation within this singular locus.

## Figures and Tables

**Table 1:** Mutation classes and frequencies for sperm and blood. Percentages are followed by number of observed mutants in parentheses. *n*: number of molecules analysed.

Mutant class	Sperm (*n* = 597)		Blood (*n* = 531)		*t-*test	
	Frequency	95% CI	Frequency	95% CI	*t*	*p*-value
All mutants	2.68 (16)	2–3.36	1.88 (11)	1.28–2.48	0.88	0.375
Length mutants	1.51 (9)	1–2.17	0.38 (2)	0.11–0.65	1.97	0.049
Isometric mutants	1.17 (7)	0.72–1.62	1.70 (9)	1.13–2.27	0.72	0.472
Modular structure mutants	0.17 (1)	0–0.34	1.70 (9)	1.13–2.27	2.56	0.011
Boundary switch mutants	1 (6)	0.6–1.4	0 (0)	0	–	–
