# Supplementary material for: Complex germline and somatic mutation processes at a haploid human minisatellite shown by single-molecule analysis
Source: Mutat Res. 2008 Dec 15;648(1-2):46–53. doi: 10.1016/j.mrfmmm.2008.09.008 (PMC2599865; doi:10.1016/j.mrfmmm.2008.09.008)
Supplement: Supplementary file 1 [file mmc1.pdf]

**Supplementary Table for Shanks et al., 'Complex germline and somatic mutation processes at a haploid human minisatellite shown by single-molecule analysis'**

| | Repeat Block | | | | | | | | | repeat | |
|---|---|---|---|---|---|---|---|---|---|---|---|
| Allele | 1 | 3 | 1 | 3 | 4 | 3 | 4 | 3 | 4 | no. | modular structure |
| (1)19 (3)39 (4)16 | | | 19 | 39 | 16 | | | | | 74 | 1,3,4 |
| (1)15 (3)41 (4)17 | | | 15 | 41 | 17 | | | | | 73 | 1,3,4 |
| (1)16(3)39(4)19 | | | 16 | 39 | 19 | | | | | 74 | 1,3,4 |
| (1)17 (3)37 (4)19 | | | 17 | 37 | 19 | | | | | 73 | 1,3,4 |
| (1)17 (3)37 (4)21 | | | 17 | 37 | 21 | | | | | 75 | 1,3,4 |
| (1)15 (3)39 (4)18 | | | 15 | 39 | 18 | | | | | 72 | 1,3,4 |
| (1)16(3)40(4)18 | | | 16 | 40 | 18 | | | | | 74 | 1,3,4 |
| (1)15 (3)38 (4)19 | | | 15 | 38 | 19 | | | | | 72 | 1,3,4 |
| (1)18(3)35(4)21 | | | 18 | 35 | 21 | | | | | 74 | 1,3,4 |
| (1)13 (3)37 (4)19 | | | 13 | 37 | 19 | | | | | 57 | 1,3,4 |
| (1)15 (3)30 (4)24 | | | 15 | 30 | 24 | | | | | 69 | 1,3,4 |
| (1)15 (3)36 (4)22 | | | 15 | 36 | 22 | | | | | 73 | 1,3,4 |
| (1)15 (3)41 (4)15 | | | 15 | 41 | 15 | | | | | 71 | 1,3,4 |
| (1)15 (3)44 (4)16 | | | 15 | 44 | 16 | | | | | 75 | 1,3,4 |
| (1)16 (3)36 (4)19 | | | 16 | 36 | 19 | | | | | 71 | 1,3,4 |
| (1)16 (3)37 (4)20 | | | 16 | 37 | 20 | | | | | 73 | 1,3,4 |
| (1)16 (3)37 (4)21 | | | 16 | 37 | 21 | | | | | 74 | 1,3,4 |
| (1)16 (3)39 (4)19 | | | 16 | 39 | 19 | | | | | 74 | 1,3,4 |
| (1)16 (3)41 (4)15 | | | 16 | 41 | 15 | | | | | 72 | 1,3,4 |
| (1)16 (3)41 (4)16 | | | 16 | 41 | 16 | | | | | 73 | 1,3,4 |
| (1)16 (3)43 (4)19 | | | 16 | 43 | 19 | | | | | 78 | 1,3,4 |
| (1)17 (3)35 (4)21 | | | 17 | 35 | 21 | | | | | 73 | 1,3,4 |
| (1)17 (3)35 (4)22 | | | 17 | 35 | 22 | | | | | 74 | 1,3,4 |
| (1)17 (3)38 (4)20 | | | 17 | 38 | 20 | | | | | 75 | 1,3,4 |
| (1)17 (3)41 (4)19 | | | 17 | 41 | 19 | | | | | 77 | 1,3,4 |
| (1)18 (3)44 (4)18 | | | 18 | 44 | 18 | | | | | 80 | 1,3,4 |
| (1)23 (3)49 (4)18 | | | 23 | 49 | 18 | | | | | 90 | 1,3,4 |
| (1)16(3)40(4)18 | | | 16 | 40 | 18 | | | | | 74 | 1,3,4 |
| (1)16(3)41(4)17 | | | 16 | 41 | 17 | | | | | 74 | 1,3,4 |
| (1)16 (3)36 (4)21 | | | 16 | 36 | 21 | | | | | 73 | 1,3,4 |
| (1)16 (3)41 (4)18 | | | 16 | 41 | 18 | | | | | 75 | 1,3,4 |
| (1)17 (3)41 (4)16 | | | 17 | 41 | 16 | | | | | 74 | 1,3,4 |
| (1)17(3)34(4)20 | | | 17 | 34 | 20 | | | | | 73 | 1,3,4 |
| (1)14 (3)39 (4)20 | | | 14 | 39 | 20 | | | | | 73 | 1,3,4 |
| (1)17 (3)40 (4)17 | | | 17 | 40 | 17 | | | | | 74 | 1,3,4 |
| (1)17(3)38(4)20 | | | 17 | 38 | 20 | | | | | 75 | 1,3,4 |
| (1)15 (3)36 (4)21 | | | 15 | 36 | 21 | | | | | 72 | 1,3,4 |
| (1)17 (3)37 (4)22 | | | 17 | 37 | 22 | | | | | 76 | 1,3,4 |
| (1)16 (3)41 (4)20 | | | 16 | 41 | 20 | | | | | 77 | 1,3,4 |
| (1)16 (3)35 (4)21 | | | 16 | 35 | 21 | | | | | 72 | 1,3,4 |
| (1)16 (3)33 (4)23 | | | 16 | 33 | 23 | | | | | 72 | 1,3,4 |
| (1)15 (3)36 (4)20 | | | 15 | 36 | 20 | | | | | 71 | 1,3,4 |
| (1)14 (3)42 (?)19 | | | 14 | 42 | 19 | | | | | 75 | 1,3,4 |
| (1)16 (3)36 (4)21 | | | 16 | 36 | 21 | | | | | 73 | 1,3,4 |
| (1)16 (3)39 (4)17 | | | 16 | 39 | 17 | | | | | 72 | 1,3,4 |
| (1)14 (3)46 (4)14 | | | 14 | 46 | 14 | | | | | 74 | 1,3,4 |
| (1)16 (3)37 (4)19 | | | 16 | 37 | 19 | | | | | 72 | 1,3,4 |
| (1)17 (3)38 (4)19 | | | 17 | 38 | 19 | | | | | 74 | 1,3,4 |
| (1)16 (3)39 (4)15 | | | 16 | 39 | 15 | | | | | 70 | 1,3,4 |
| (1)15 (3)42 (4)17 | | | 15 | 42 | 17 | | | | | 74 | 1,3,4 |
| (1)15 (3)42 (4)18 | | | 15 | 42 | 18 | | | | | 75 | 1,3,4 |
| (1)15 (3)37 (4)20 | | | 15 | 37 | 20 | | | | | 72 | 1,3,4 |
| (1)15 (3)39 (4)19 | | | 15 | 39 | 19 | | | | | 72 | 1,3,4 |
| (1)15 (3)39 (4)19 | | | 15 | 39 | 19 | | | | | 73 | 1,3,4 |
| (1)15 (3)39 (4)19 | | | 15 | 39 | 19 | | | | | 73 | 1,3,4 |

| Allele | Repeat Block 1 | 3 | 1 | 3 | 4 | 3 | 4 | 3 | 4 | repeat no. | modular structure |
|---|---|---|---|---|---|---|---|---|---|---|---|
| (1)15 (3)40 (4)16 | | | 15 | 40 | 16 | | | | | 71 | 1,3,4 |
| (1)15 (3)41 (4)14 | | | 15 | 41 | 14 | | | | | 70 | 1,3,4 |
| (1)15 (3)41 (4)14 | | | 15 | 41 | 14 | | | | | 70 | 1,3,4 |
| (1)15 (3)41 (4)15 | | | 15 | 41 | 15 | | | | | 71 | 1,3,4 |
| (1)15 (3)41 (4)15 | | | 15 | 41 | 15 | | | | | 71 | 1,3,4 |
| (1)15 (3)41 (4)15 | | | 15 | 41 | 15 | | | | | 71 | 1,3,4 |
| (1)15 (3)41 (4)18 | | | 15 | 41 | 18 | | | | | 74 | 1,3,4 |
| (1)15 (3)41 (4)20 | | | 15 | 41 | 20 | | | | | 76 | 1,3,4 |
| (1)15 (3)41 (4)23 | | | 15 | 41 | 23 | | | | | 79 | 1,3,4 |
| (1)15 (3)41 (4)23 | | | 15 | 41 | 23 | | | | | 79 | 1,3,4 |
| (1)15 (3)42 (4)15 | | | 15 | 42 | 15 | | | | | 72 | 1,3,4 |
| (1)15 (3)42 (4)15 | | | 15 | 42 | 15 | | | | | 72 | 1,3,4 |
| (1)15 (3)42 (4)16 | | | 15 | 42 | 16 | | | | | 73 | 1,3,4 |
| (1)15 (3)42 (4)16 | | | 15 | 42 | 16 | | | | | 73 | 1,3,4 |
| (1)15 (3)42 (4)16 | | | 15 | 42 | 16 | | | | | 73 | 1,3,4 |
| (1)15 (3)42 (4)16 | | | 15 | 42 | 16 | | | | | 73 | 1,3,4 |
| (1)15 (3)42 (4)17 | | | 15 | 42 | 17 | | | | | 74 | 1,3,4 |
| (1)15 (3)42 (4)18 | | | 15 | 42 | 18 | | | | | 75 | 1,3,4 |
| (1)15 (3)42 (4)18 | | | 15 | 42 | 18 | | | | | 75 | 1,3,4 |
| (1)16 (3)36 (4)20 | | | 16 | 36 | 20 | | | | | 72 | 1,3,4 |
| (1)16 (3)37 (4)21 | | | 16 | 37 | 21 | | | | | 74 | 1,3,4 |
| (1)16 (3)38 (4)21 | | | 16 | 38 | 21 | | | | | 75 | 1,3,4 |
| (1)16 (3)39 (4)15 | | | 16 | 39 | 15 | | | | | 70 | 1,3,4 |
| (1)16 (3)39 (4)20 | | | 16 | 39 | 20 | | | | | 75 | 1,3,4 |
| (1)16 (3)40 (4)16 | | | 16 | 40 | 16 | | | | | 72 | 1,3,4 |
| (1)16 (3)40 (4)18 | | | 16 | 40 | 18 | | | | | 74 | 1,3,4 |
| (1)16 (3)44 (4)16 | | | 16 | 44 | 16 | | | | | 76 | 1,3,4 |
| (1)16 (3)46 (4)13 | | | 16 | 46 | 13 | | | | | 75 | 1,3,4 |
| (1)18 (3)38 (4)18 | | | 18 | 38 | 18 | | | | | 74 | 1,3,4 |
| (1)18 (3)38 (4)18 | | | 18 | 38 | 18 | | | | | 74 | 1,3,4 |
| (1)16 (3)40 (4)17 | | | 16 | 40 | 17 | | | | | 73 | 1,3,4 |
| (1)16 (3)40 (4)18 | | | 16 | 40 | 18 | | | | | 74 | 1,3,4 |
| (1)16 (3)37 (4)18 | | | 16 | 37 | 18 | | | | | 71 | 1,3,4 |
| (1)16 (3)37 (4)19 | | | 16 | 37 | 19 | | | | | 72 | 1,3,4 |
| (1)16 (3)38 (4)20 | | | 16 | 38 | 20 | | | | | 74 | 1,3,4 |
| (1)12 (3)44 (4)19 | | | 12 | 44 | 19 | | | | | 75 | 1,3,4 |
| (1)16 (3)37 (4)19 | | | 16 | 37 | 19 | | | | | 72 | 1,3,4 |
| (1)15 (3)37 (4)20 | | | 15 | 37 | 20 | | | | | 72 | 1,3,4 |
| (1)16 (3)39 (4)20 | | | 16 | 39 | 20 | | | | | 75 | 1,3,4 |
| (1)16 (3)38 (4)20 | | | 16 | 38 | 20 | | | | | 74 | 1,3,4 |
| (1)17 (3)37 (4)15 | | | 17 | 37 | 15 | | | | | 69 | 1,3,4 |
| (1)16 (3)40 (4)18 | | | 16 | 40 | 18 | | | | | 74 | 1,3,4 |
| (1)15 (3)36 (4)21 | | | 15 | 36 | 21 | | | | | 72 | 1,3,4 |
| (1)17 (3)37 (4)19 | | | 17 | 37 | 19 | | | | | 73 | 1,3,4 |
| (1)16 (3)37 (4)18 | | | 16 | 37 | 18 | | | | | 71 | 1,3,4 |
| (1)17 (3)37 (4)20 | | | 17 | 37 | 20 | | | | | 74 | 1,3,4 |
| (1)16 (3)41 (4)18 | | | 16 | 41 | 18 | | | | | 75 | 1,3,4 |
| (1)12 (3)46 (4)17 | | | 12 | 46 | 17 | | | | | 75 | 1,3,4 |
| (1)16 (3)38 (4)20 | | | 16 | 38 | 20 | | | | | 74 | 1,3,4 |
| (1)15 (3)37 (4)21 | | | 15 | 37 | 21 | | | | | 73 | 1,3,4 |
| (1)15 (3)39 (4)19 | | | 15 | 39 | 19 | | | | | 73 | 1,3,4 |
| (1)15 (3)40 (4)19 | | | 15 | 40 | 19 | | | | | 74 | 1,3,4 |
| (1)16 (3)34 (4)22 | | | 16 | 34 | 22 | | | | | 72 | 1,3,4 |
| (1)16 (3)34 (4)22 | | | 16 | 34 | 22 | | | | | 72 | 1,3,4 |
| (1)16 (3)38 (4)21 | | | 16 | 38 | 21 | | | | | 75 | 1,3,4 |
| (1)17 (3)38 (4)19 | | | 17 | 38 | 19 | | | | | 74 | 1,3,4 |
| (1)15 (3)40 (4)19 | | | 15 | 40 | 19 | | | | | 74 | 1,3,4 |
| (1)16 (3)41 (4)18 | | | 16 | 41 | 18 | | | | | 75 | 1,3,4 |
| (1)15 (3)38 (4)20 | | | 15 | 38 | 20 | | | | | 73 | 1,3,4 |
| (1)16 (3)39 (4)18 | | | 16 | 39 | 18 | | | | | 73 | 1,3,4 |
| (1)16 (3)40 (4)19 | | | 16 | 40 | 19 | | | | | 75 | 1,3,4 |
| (1)16 (3)40 (4)18 | | | 16 | 40 | 18 | | | | | 74 | 1,3,4 |

| | Repeat Block | | | | | | | | | repeat | |
|---|---|---|---|---|---|---|---|---|---|---|---|
| **Allele** | **1** | **3** | **1** | **3** | **4** | **3** | **4** | **3** | **4** | **no.** | **modular structure** |
| (1)14 (3)39 (4)19 | | | 14 | 39 | 19 | | | | | 72 | 1,3,4 |
| (1)16 (3)41 (4)17 | | | 16 | 41 | 17 | | | | | 74 | 1,3,4 |
| (1)16 (3)38 (4)19 | | | 16 | 38 | 19 | | | | | 73 | 1,3,4 |
| (1)16 (3)38 (4)19 | | | 16 | 38 | 19 | | | | | 73 | 1,3,4 |
| (1)17 (3)37 (4)19 | | | 17 | 37 | 19 | | | | | 73 | 1,3,4 |
| (1)15 (3)39 (4)18 | | | 15 | 39 | 18 | | | | | 72 | 1,3,4 |
| (1)16 (3)41 (4)16 | | | 16 | 41 | 16 | | | | | 73 | 1,3,4 |
| (1)16 (3)39 (4)19 | | | 16 | 39 | 19 | | | | | 74 | 1,3,4 |
| (1)16 (3)38 (4)19 | | | 16 | 38 | 19 | | | | | 73 | 1,3,4 |
| (1)16 (3)38 (4)19 | | | 16 | 38 | 19 | | | | | 73 | 1,3,4 |
| (1)16 (3)38 (4)18 | | | 16 | 38 | 18 | | | | | 72 | 1,3,4 |
| (1)16 (3)41 (4)15 | | | 16 | 41 | 15 | | | | | 72 | 1,3,4 |
| (1)16 (3)38 (4)18 | | | 16 | 38 | 18 | | | | | 72 | 1,3,4 |
| (1)16 (3)38 (4)18 | | | 16 | 38 | 18 | | | | | 72 | 1,3,4 |
| (1)16 (3)38 (4)17 | | | 16 | 38 | 17 | | | | | 71 | 1,3,4 |
| (1)16 (3)38 (4)19 | | | 16 | 38 | 19 | | | | | 73 | 1,3,4 |
| (1)17 (3)38 (4)14 | | | 17 | 38 | 14 | | | | | 69 | 1,3,4 |
| (1)16 (3)38 (4)17 | | | 16 | 38 | 17 | | | | | 71 | 1,3,4 |
| (1)18 (3)39 (4)19 | | | 18 | 39 | 19 | | | | | 76 | 1,3,4 |
| (1)16 (3)44 (4)14 | | | 16 | 44 | 14 | | | | | 74 | 1,3,4 |
| (1)16 (3)44 (4)16 | | | 16 | 44 | 16 | | | | | 76 | 1,3,4 |
| (1)16 (3)38 (4)19 | | | 16 | 38 | 19 | | | | | 73 | 1,3,4 |
| (1)16 (3)38 (4)19 | | | 16 | 38 | 19 | | | | | 73 | 1,3,4 |
| (1)16 (3)41 (4)16 | | | 16 | 41 | 16 | | | | | 73 | 1,3,4 |
| (1)17 (3)37 (4)19 | | | 17 | 37 | 19 | | | | | 73 | 1,3,4 |
| (1)16 (3)39 (4)18 | | | 16 | 39 | 18 | | | | | 73 | 1,3,4 |
| (1)18 (3)38 (4)17 | | | 18 | 38 | 17 | | | | | 73 | 1,3,4 |
| (1)14 (3)38 (4)18 | | | 14 | 38 | 18 | | | | | 70 | 1,3,4 |
| (1)17 (3)38 (4)1 (3)3 (4)16 | | | 17 | 38 | 1 | 3 | 16 | | | 75 | 1,3,4,3,4 |
| (1)17 (3)35 (4)2 (3)1 (4)22 | | | 17 | 35 | 2 | 1 | 22 | | | 77 | 1,3,4,3,4 |
| (1)16 (3)39 (4)1 (3)1 (4)19 | | | 16 | 39 | 1 | 1 | 19 | | | 76 | 1,3,4,3,4 |
| (1)19 (3)35 (4)1 (3)1 (4)16 | | | 19 | 35 | 1 | 1 | 16 | | | 72 | 1,3,4,3,4 |
| (1)15 (3)38 (4)2 (3)1 (4)15 | | | 15 | 38 | 2 | 1 | 15 | | | 71 | 1,3,4,3,4 |
| (1)16 (3)37 (4)3 (3)2 (4)2 (3)2 (4)12 | | | 16 | 37 | 3 | 2 | 2 | 2 | 12 | 74 | 1,3,4,3,4,3,4 |
| (1)16 (3)38 (4)3 (3)2 (4)2 (3)2 (4)12 | | | 16 | 37 | 3 | 2 | 2 | 2 | 12 | 75 | 1,3,4,3,4,3,4 |
| (1)16 (3)38 (4)3 (3)2 (4)2 (3)2 (4)12 | | | 16 | 38 | 3 | 2 | 2 | 2 | 12 | 75 | 1,3,4,3,4,3,4 |
| (1)16 (3)38 (4)3 (3)2 (4)2 (3)3 (4)11 | | | 16 | 38 | 3 | 2 | 2 | 3 | 11 | 75 | 1,3,4,3,4,3,4 |
| (1)17 (3)38 (4)1 (3)3 (4)1 (3)4 (4)10 | | | 17 | 38 | 1 | 3 | 1 | 4 | 10 | 74 | 1,3,4,3,4,3,4 |
| (1)16 (3)39 (4)1 (3)3 (4)2 (3)2 (4)12 | | | 16 | 39 | 1 | 3 | 2 | 2 | 12 | 75 | 1,3,4,3,4,3,4 |
| (1)18 (3)5 (1/0)1 (3)37 (4)21 | 18 | 5 | 1 | 37 | 21 | | | | | 82 | 1,3,1,3,4 |
| (1)18 (3)8 (1)1 (3)36 (4)20 | 18 | 8 | 1 | 36 | 20 | | | | | 83 | 1,3,1,3,4 |
| (1)16 (3)2 (1)1 (3)40 (4)1 (3)2 (4)15 | 16 | 2 | 1 | 40 | 1 | 2 | 15 | | | 77 | 1,3,1,3,4,3,4 |
| | **1** | **3** | **1** | **3** | **4** | **3** | **4** | **3** | **4** | | |
